# Indigenously developed multipurpose acrylic head phantom for verification of IMRT using film and gel dosimetry

**DOI:** 10.1120/jacmp.v14i2.4041

**Published:** 2013-03-04

**Authors:** N. Gopishankar, S. Vivekanandhan, G.K. Rath, M.A. Laviraj, S. Senthilkumaran, S.S. Kale, S. Thulkar, R.K. Bisht, V. Subramani

**Affiliations:** ^1^ Gamma Knife Unit, Department of Neurosurgery Neurosciences Centre; ^2^ Department of Neurobiochemistry Neurosciences Centre; ^3^ Dept of Radiation Oncology Dr. B. R. Ambedkar Institute Rotary Cancer Hosptial; ^4^ Department of Nuclear Magnetic Resonance; ^5^ Department of Radio Diagnosis All India Institute of Medical Sciences Ansari Nagar New Delhi India

**Keywords:** acrylic phantom, IMRT, MAGAT gel, EBT2 film, gamma index analysis

## Abstract

The purpose of this study was to validate the newly designed acrylic phantom for routine dosimetric purpose in radiotherapy. The phantom can be used to evaluate and compare the calculated dose and measured dose using film and gel dosimetric methods. In this study, a doughnut‐shaped planning target volume ($8.54\;{\text{cm}}^{3}$) and inner organ at risk ($0.353\;{\text{cm}}^{3}$) were delineated for an IMRT test plan using the X‐ray CT image of the phantom. The phantom consists of acrylic slabs which are integrated to form a human head with a hole in the middle where several dosimetric inserts can be positioned for measurement. An inverse planning with nine coplanar intensity‐modulated fields was created using Pinnacle TPS. For the film analysis, EBT2 film, flatbed scanner, in‐house developed MATLAB codes and ImageJ software were used. The 3D dose distribution recorded in the MAGAT gel dosimeter was read using a 1.5 T MRI scanner. Scanning parameters were CPMG pulse sequence with 8 equidistant echoes, TR=5600, echo step=22 ms, pixel size=0.5 times 0.5, slice thickness=2 mm. Using a calibration relationship between absorbed dose and spin‐spin relaxation rate (R2), R2 images were converted to dose images. The dose comparison was accomplished using in‐house MATLAB‐based graphical user interface named “IMRT3DCMP”. For gel measurement dose grid from the TPS was extracted and compared with the measured dose grid of the gel. Gamma index analysis of film measurement for the tolerance criteria of 2%/2 mm, 1%/1 mm showed more than 90% voxels pass rate. Gamma index analysis of 3D gel measurement data showed more than 90% voxels pass rate for different tolerance criteria of 2%/2 mm and 1%/1 mm. Overall both 2D and 3D measurement were in close agreement with the Pinnacle TPS calculated dose. The phantom designed is cost‐effective and the results are promising, but further investigation is required to validate the phantom with other 3D conformal techniques for dosimetric purpose.

PACS numbers: 87.53.Kn, 87.55.km, 87.56.N‐

## I. INTRODUCTION

In the recent years, gel dosimetry is demonstrating its potential to be a very useful tool for three‐dimensional visualization and measurement of absorbed dose distribution in a variety of medical applications such as stereotactic radiosurgery (SRS), intensity‐modulated radiotherapy (IMRT), brachytherapy, and breathing adapted radiotherapy (BART). Interestingly all these applications use small radiation fields for treatment. The most vital problems present in all conventional dosimetric systems are perturbation effects, volume averaging, and positioning (setup errors).^(^ ^1^ ^,^ ^2^ ^,^ ^3^ ^)^ It is believed that these problems can be addressed using polymer gel dosimetry, which is a dosimetric method whereby the dosimeter and the phantom are identical and practically water equivalent.^(^ ^2^ ^,^ ^4^ ^,^ ^5^ ^)^ Most authors sought to model the real‐world situation using appropriate shapes and dimensions of gel phantoms; however, the flexibility of gel dosimeters enables one to manufacture more authentic phantoms.^(^ ^6^ ^)^ There are several commercial phantoms available in market. For example, the Alderson RANDO phantom has been in use for over 30 years (Radiology Support Devices Inc, Long Beach, CA). Some of the disadvantages of this phantom are it is costly and bulky, and it is a tedious process to shift from one machine to another to perform dosimetry. Lucy 3D QA phantom (Standard Imaging Inc., Middleton, WI) has been widely used for dosimetric purpose. It is spherical in shape which is suitable for dosimetry in ideal conditions, but it lacks the true shape of the patient's head which is necessary for studying the effect of patient's skull geometry while performing dosimetry. The Radiological Physics Center (RPC) at the MD Anderson Cancer Center (University of Texas, Houston, TX) has designed and constructed four phantoms to evaluate advanced treatment technologies that represent the brain, head and neck, the thorax, and pelvis.^(^ ^7^ ^,^ ^8^ ^)^ RPC phantoms have been designed to perform dosimetric audits for several institutions. Although it is feasible to do such studies internationally, it is highly labor‐intensive. A dedicated acrylic phantom which is lightweight, easily set up in a treatment unit for dosimetry, specifically for performing 3D measurements with gel dosimeters, was designed in‐house to simulate the shape of the head and neck region.

This work presents an investigation of the feasibility of gel dosimetry with in‐house made acrylic head phantom. The phantom and the software tools developed have promising features for performing IMRT credential test for institutions participating in clinical trial protocols. Although IMRT is an advanced type of high‐precision radiation treatment, currently available conventional dosimeters for radiation measurement are point, 2D, 3D array of detectors, and scintillation devices, which lack the true three‐dimensional measurement capability. In this work, we demonstrate the role of radiation‐sensitive gel as a detector which has the high potential for comprehensive 3D measurements. MAGAT gel was used for this 3D verification study, which is a modified version of the first normoxic polymer gel named MAGIC gel.^(^ ^9^ ^)^ MAGAT gel has the advantage over MAGIC gel in tissue equivalence and dose sensitivity.^(^ ^10^ ^)^ Several research groups and commercial vendors are showing keen interest to establish 3D dosimetry in radiotherapy clinic. As far as IMRT is concerned, one of the key components as listed by Moran et al.^(^ ^11^ ^)^ in their study is to perform patient specific pretreatment QA. For this purpose, some of the centers have developed their own techniques to improve QA efficiency. Through this work we have developed tools which were already tested for 3D measurements to perform measurements and analyses in a radiation oncology clinic using normoxic gels.^(^ ^12^ ^,^ ^13^ ^,^ ^14^ ^)^ We have used the same tools with minor modifications for verification of IMRT plan.

## II. MATERIALS AND METHODS

### A. Phantom design

**Figure 1:**
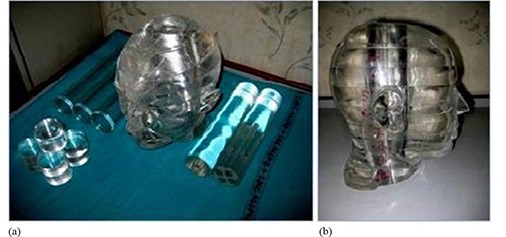
A multipurpose head phantom design (a) for dosimetric purpose. The figure also shows several dosimetric inserts. Three cylinders on the right side of the phantom are for gel measurement, small discs are for film measurement, and two cylinders on the left side of phantom are for ion chamber measurements. Sagittal view (b) of the acrylic phantom.

An acrylic phantom with human head shape was designed for gel dosimetry purpose, as shown in Fig. 1. In addition it has the potential to be used for film dosimetry, ion chamber measurement, and TLD measurement. The phantom consists of acrylic slabs which are integrated to form a human head shaped with a hole in the middle where several dosimetric inserts can be positioned for measurement. Several cylindrical insert with length 24 cm and 5 cm diameter were designed for dosimetric purpose. Three hollow cylinders were designed specifically for gel measurement purpose. The gel inserts have an inner diameter of 4.6 cm. For film measurement, a single solid cylinder was cut into small discs with a region for holding GAFCHROMIC film (International Specialty Products, Wayne, NJ) (see Fig. 1).

### B. Gel preparation

For the gel preparation, gelatin was soaked in water for 1 hr. The contents were stirred thoroughly in a water bath overhead stirrer assembly at 50°C till a clear solution was obtained. The temperature was brought down to 38°C and hydroquinone with methacrylic acid was added and the contents were stirred further. Finally tetrakis(hydroxymethyl)phosphonium chloride (THPC) was added to the gel composition by bringing down the temperature to 20°C.^(^ ^9^ ^,^ ^15^ ^,^ ^16^ ^)^ The prepared gel was poured in to three cylindrical gel inserts which were used for IMRT plan verification and into small gel vials or tubes (see Table 1).

**Table 1:** MAGAT gel composition.

	*Composition*
Gelatin	6%
Methacrylic Acid	9%
THPC	10 mM
$H_{2}$O	83%
Hydroquinone	0.05 mM
Gel Volume	1500 mL
Dose Range	0–5 Gy

### C. Gel calibration

Calibration of gel for IMRT study was carried out with seven acrylic tubes of 15 cm length, 2 cm outer diameter, 1.5 cm inner diameter. Six vials were used for calibration to doses from 50 cGy to 500 cGy. One vial was used as control vial. The tubes were placed in water tank and irradiated with parallel‐opposed 6 MV photons from SynergyS (Elekta, Stockholm, Sweden) with field size of $16\times 16\;{\text{cm}}^{2}$.

### D. Gel irradiation for an experimental case

**Figure 2:**
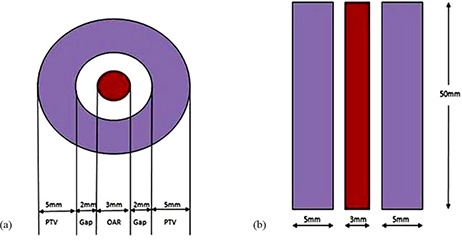
Axial view (a) of a doughnut‐shaped planning target volume (PTV) and inner organ at risk (OAR) in the computed tomography image. Coronal view (b) of the PTV with length of 50 mm created in X‐ray CT image.

X‐ray CT images were obtained of the phantom with cylindrical acrylic insert with scanning parameters: 120 k V, 400 mA, slice thickness 1.5 mm, matrix size 512×512, field of view 250 mm. The phantom CT images were pushed to ADAC Pinnacle planning system (Philips Healthcare, Andover, MA) for creating an experimental plan (see Fig. 2).

**Figure 3:**
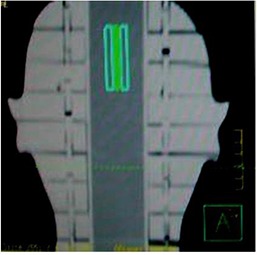
An experimental target created in the phantom which is a doughnut‐shaped cylinder (coronal view).

A doughnut‐shaped target volume ($8.54\;{\text{cm}}^{3}$) and inner organ at risk ($0.353\;{\text{cm}}^{3}$) were delineated for an IMRT test planning using the X‐ray CT image of the phantom (see Figs. 2 and 3). This target resembles a spinal metastasis target. A nine‐field IMRT plan (${\text{Pinnacle}}^{3}$ Version ADAC, 8.0M, Philips Medical System) was created.^(^ ^17^ ^)^ A calculation grid of 2 mm was used. The phantom with gel insert filled with MAGAT gel was positioned in the treatment couch and exposed to the planned irradiation. A daily fraction dose of 1.5 Gy was delivered to the planning target volume as it was an experimental study. After irradiation, the phantom was left in the MRI scanning room for approximately 24 hrs before scanning to attain thermal equilibrium.

### E. Gel scanning

The irradiated gels were scanned with a 1.5 Tesla Siemens Sonata MR imager (Siemens Medical Systems, Erlangen, Germany) using a slice selective 8‐echo Carr‐Purcell‐Meiboom‐Gill sequence (CPMG) with an initial echo time of 22 ms, with further increments of 22 ms and a repetition time of 5.6 s. The slice thickness was selected to be 2 mm without gap between slices; field of view=256 mm, matrix size=512×512, and $\text{pixel}\;\text{size}=0.5\times 0.5\;{\text{mm}}^{2}$. The acrylic tubes for calibration were scanned together with the head phantom. Three Vitamin E capsules were used to mark the isocenter in the MRI to allow the registration of the measured dose distribution images with the planned dose distribution exported by TPS.

We used an in‐house MATLAB program‐based GUI routine “ATOM” (The MathWorks, Natick, MA) which processes the DICOM data generated by a MRI scanner and computes the R2 maps using the VAREC method.^(^ ^18^ ^)^ A calibration relationship between R2 (spin‐spin relaxation rate) and absorbed dose using the images of the gel vials was obtained. Then the R2 images of the phantom with acrylic cylindrical insert and vials were converted to dose images by applying the calibration equation.^(^ ^18^ ^,^ ^19^ ^,^ ^20^ ^,^ ^21^ ^)^ It is noted that the median image filter was applied to all raw MR image data before the R2 estimation algorithm was used in the ATOM program.

### F. Film irradiation and measurement

For film analysis, EBT2 GAFCHROMIC films were cut into small circular strips of 4.5 cm diameter and placed in the film insert for irradiation, per the planning (see Materials and Methods Section D). For calibration, films from the same batch were cut into small strips and exposed to different doses, from 0 to 5 Gy, using acrylic slab phantom. Film orientation was preserved by making pricks on the film. A flatbed scanner (Microtek 9800XL; Microtek Lab Inc., Santa Fe Springs, CA) was used to scan the exposed films in transmission mode. After a scanner warming time of 30 minutes, the calibration and measurement films were scanned five times to minimize the random noises and uncertainties that occurred during the scanning procedures. All the films were positioned in the middle region of the flatbed scanner to avoid uncertainty in calibration. The film images were split into three color channels — red, green, and blue. The pixel values obtained from each channel was converted into optical density (OD) and calibration curve of the second degree polynomial was determined for each channel using curve fitting function of free download ImageJ software. Detailed film dosimetry procedure is described in Devic et al. and others.^(^ ^22^ ^,^ ^23^ ^,^ ^24^ ^,^ ^25^ ^)^ We applied similar procedures for our study. The obtained dose map was compared with TPS‐calculated dose grid using in‐house developed MATLAB codes.

### G. Analysis tools for comparison

For the comparison of experimental data derived with corresponding TPS calculations, the raw data (TPS‐calculated) in a 3D grid suitable for comparison with the 3D matrix of experimental relative dose data were extracted from the TPS and imported into in‐house MATLAB‐based GUI program IMRT3DCMP. The Pinnacle TPS dose grid for the whole acrylic phantom was of matrix size $236\times 236\times 39\;{\text{mm}}^{3}$ (voxel size 1 mm×1 mm×2 mm), and the MRI measured dose map was a matrix of size $512\times 512\times 39{\text{mm}}^{3}$ (voxel size 0.5 mm×0.5 mm×2 mm). TPS dose grid and the MRI‐measured gel dose were entered in the GUI for analysis, which first recomputed the measured dose values at the 3D spatial grid points of the computed dose matrix ((236×236×39=2172144 voxels)) by using a 3D interpolation algorithm “interp3” routine in MATLAB. Using measured and calculated dose matrices, dose differences at all points in the dose matrix were calculated. The dose difference was defined as a subtraction of the calculated dose from the measured dose.^(^ ^12^ ^,^ ^13^ ^,^ ^26^ ^)^ The dose difference values were grouped based on the dose difference and the dose level of the Pinnacle TPS calculation at that point. Three‐dimensional dose comparisons between calculated and measured dose were made by plotting isodose overlay, dose differences or the gamma evaluation on planes.^(^ ^27^ ^)^ For the gel study, gamma evaluation criteria with varying spatial tolerance (1−2 mm) and dosimetric tolerance (1%−2%) were chosen.

## III. RESULTS

### A. Film measurement results

**Figure 4:**
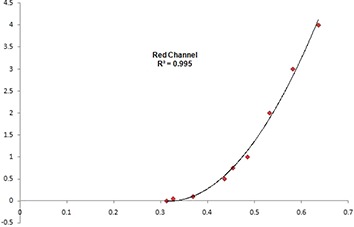
A film calibration plot between optical density (OD) and dose (cGy).

**Figure 5:**
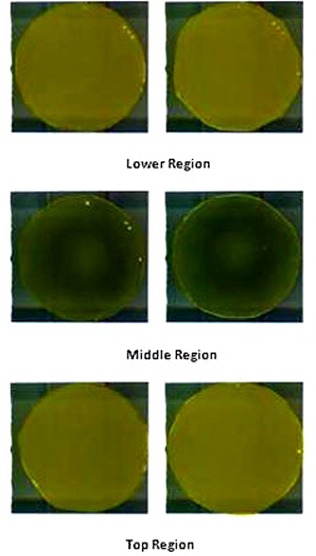
Nine‐field coplanar IMRT field exposure in EBT2 GAFCHROMIC film using SynergyS linac at different positions of the phantom.

Figure 4 shows the calibration plot between optical density (OD) and dose (cGy) measured in GAFCHROMIC EBT2 film. A second degree polynomial fit was made for the calibration plot. Six films shown in Fig. 5 were exposed to nine‐field IMRT technique. The films show exposure in three different regions of the phantom. The dose exposure is more prominent in the mid‐region. Pair of films was used at different regions.

**Figure 6:**
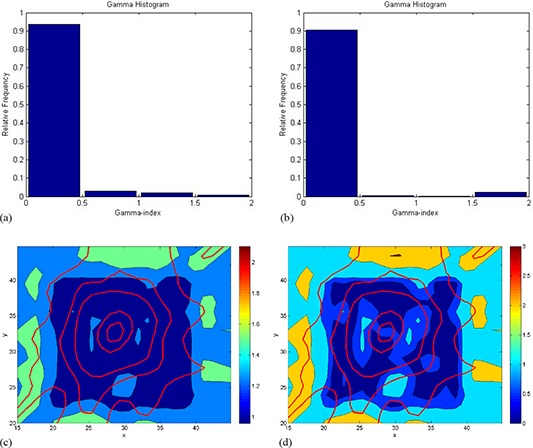
Histogram of gamma evaluation map for different pass criteria of film measurement: (a) 2%/2 mm, (b) 1%/ 1 mm. Gamma index distributions (c) plotted on transverse plane for film analysis of tolerance criteria: (c) 2%/2 mm, (d) 1%/1 mm.

In gamma index analysis histogram for gamma (2%, 2 mm), 94% of the pixels had a gamma value less than unity (see Fig. 6(a). For gamma (1%, 1 mm), 91% of the pixels had gamma value less than unity, as shown in Fig. 6(b). Gamma maps of the film comparison shows the gamma analysis in a more detailed way. In Figs. 6(c) and 6(d), the blue colored region indicates more than 90% of the pixels have gamma value less than unity. As the tolerance criteria became stricter, the dark blue color in Fig. 6(c) is changed to light blue color (see Fig. 6(d). The red lines in both gamma maps represent isodose lines of the calculated TPS dose. Green color and yellow color patches in the gamma maps shown in Figs. 6(c) and 6(d), respectively, indicate gamma value more than unity as these regions are at the edges of the GAFCHROMIC film beyond which measurement was not possible. This was due to the dimension limitations of the cylindrical insert. Figure 6(e) shows the isodose overlay comparison between the calculated TPS dose and the measured film dose. Isodose curves 1.7 Gy and 2 Gy show close agreement, whereas 1 Gy isodose curve shows a fair agreement as most of this isodose curve's path is out of film measurement region.

### B. Gel measurement results

**Figure 7:**
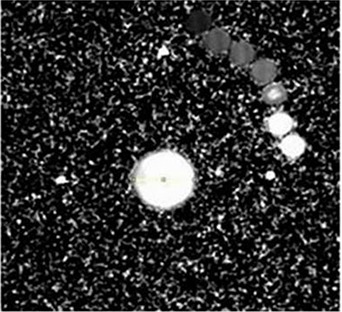
The R2 map of the phantom with cylindrical gel insert and seven acrylic vials. Three distinct small white spots indicate the fiducial points created using Vitamin E capsules.

Figure 7 depicts the R2 map of the phantom with cylindrical gel insert and seven acrylic vials. Three distinct small white spots indicate the fiducial points created using Vitamin E capsules. The phantom structure is not visible as it is a solid acrylic phantom.

The gel vials with treatment exposed cylindrical insert were left in the scanning room for more than 10 hrs to attain room temperature. The calibration plot made between R2 and dose showed a linear response.

The IMRT3DCMP program was used to plot the slice‐by‐slice isodose distributions calculated the percentage dose differences, dose‐volume histogram (DVH), differential dose‐volume histogram (DDVH), difference in differential dose‐volume histogram (DDDVH), and the gamma index values.

For all this analysis, evaluation region was restricted to a volume which corresponds to 50 times 50 times $22{\text{mm}}^{3}$. This is to be done because the gel container occupies only a smaller portion of the whole phantom. The calculated and measured 3D dose distributions were compared by interpolating them in a common space with $1.0\times 1.0\times 1.0\;{\text{mm}}^{3}$ grids (see Materials and Methods Section G).

**Figure 8:**
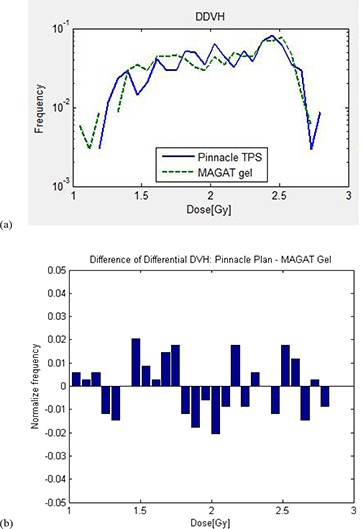
Differential dose‐volume histogram (DDVH) (a) and difference of differential dose‐volume histogram (DDDVH) (b) showing comparison of MAGAT measurements (green line) and Pinnacle calculations (blue line).

**Figure 9:**
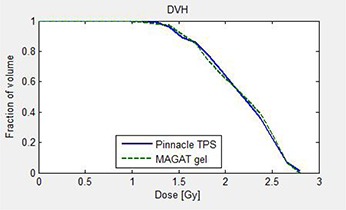
Dose‐volume histogram (right panel) showing comparison of MAGAT measurements (green line) and Pinnacle calculations (blue line).

Figure 8(a) depicts the differential dose‐volume histogram (DDVH), which indicates the (relative) number of voxels with specific dose for the measured and calculated dose.^(^ ^12^ ^)^ A reasonable agreement is observed in the comparison even though the target chosen for this study is small in dimension. The difference in differential dose‐volume histogram (DDDVH) shown in Fig. 8(b) was obtained by subtracting the number of voxels in dose bins for the measured dose from those for the calculated dose. This figure clearly shows that the measured dose was higher than the calculation in the following dose ranges: between 1 Gy and 1.2 Gy, between 1.45 Gy and 1.75 Gy, between 2.15 Gy and 2.2 Gy, and between 2.5 Gy and 2.6 Gy. For the dose between 1.8 Gy and 2.1 Gy, measured dose was significantly smaller than the calculated dose, although it was smaller at other regions, as well. The histogram bars below zero in y scale of Fig. 8(b) indicate measured dose is smaller than calculated dose at particular dose levels. Figure 9 is the dose‐volume histogram (DVH) comparison between the gel‐measured dose (green line) and TPS dose (blue line), which is another way of representing our results. It shows that comparison is matching reasonably well in the region of measurement. Minor deviations between the DVH of measured dose and calculated dose may be due to initial differences in matrix resolution of both the dose data. Please note that interpolation was done to acquire the measured gel dose and calculated TPS dose in same resolution which could add to the uncertainties (see Materials and Methods Section G).

**Figure 10:**
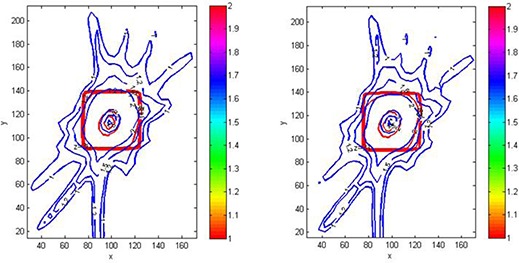
Comparison of MAGAT‐measured (red line) and Pinnacle‐calculated (blue line) dose distributions plotted on transverse planes: (a) Z=92.2 mm, (b) Z=93.76 mm. Color bar indicates the dose values in Gy.

**Figure 11:**
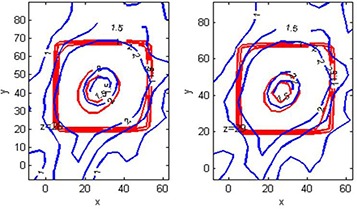
Enlarged view of comparison of MAGAT‐measured (red line) and Pinnacle‐calculated (blue line) dose distributions plotted on transverse planes as shown in Fig. 10.

**Figure 12:**
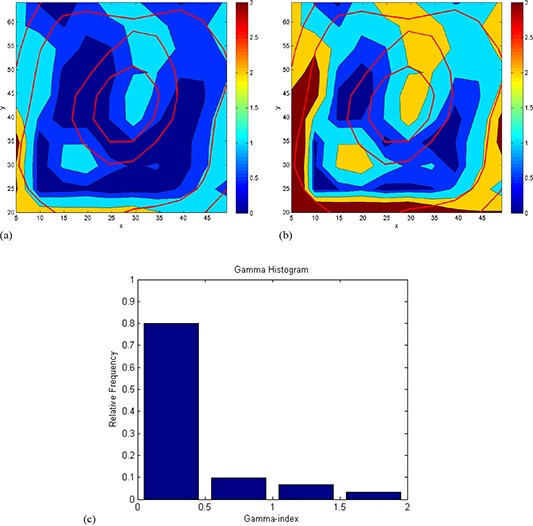
Gamma index distributions plotted on transverse plane at Z=92.2 mm for gel measurement analysis. The criteria for the gamma index calculations were: (a) 1% dose difference and 1 mm distance‐to‐agreement, (b) 2% dose difference and 2 mm distance‐to‐agreement. The units of x‐ and y‐axes are in mm. Histogram (c) showing the gamma index distribution for the whole 3D data. The criteria for the gamma index calculations were 1% dose difference and 1 mm distance‐to‐agreement.

The isodose overlay of MAGAT‐measured (solid line) and Pinnacle‐calculated (thin line) dose distributions plotted on transverse planes (a) Z=92.2 mm, (b) Z=93.76 mm showed reasonable agreement, as shown in Fig. 10. For clarity purpose only 1.5 Gy and 2 Gy isodose line is shown. Figure 11 is the magnified version of Fig. 10. Gamma index plots of corresponding slice Z=92.2 mm for tolerance criteria of 2%/2 mm and 1%/1 mm are shown in Figs. 12(a) and 12(b), respectively. The gamma index is smaller than 1 for the majority of the high‐dose region. For tolerance criteria of 2%/2 mm, 1% 1 mm, gamma pass rate was above 92% and 89%, respectively. The gamma index analysis can be summarized by using a histogram, as shown in Fig. 12(c), which indicates the portion of the voxels meeting the tolerance criteria. For the tolerance criteria of 1%/1 mm, about 89% of voxels included in the gamma index calculations had γ smaller than unity.

**Figure 13:**
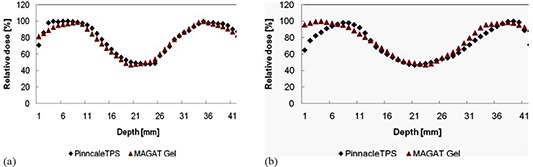
Comparison of calculated TPS dose with measured gel dose presented by horizontal (a) and vertical (b) dose profiles. The blue diamond symbol indicates the Pinnacle TPS dose and the red triangle indicates the MAGAT gel measured dose.

To show the comparison in a better manner, dose profiles were taken in horizontal and vertical directions in one particular image position out of the whole 3D data. The measured MAGAT gel dose followed the peak and valley of the calculated Pinnacle TPS dose reasonably. Main outcome of the profile study is that, in the middle region of the doughnut‐shaped target where there is rapid fall of dose, we obtained a good agreement between the calculated and measured dose. It is represented by the valley in the profile (see Figs. 13(a) and (b)).

## IV. DISCUSSION

Through this work, a 3D verification of IMRT delivered dose was made possible with the in‐house made phantom. In‐house developed MATLAB codes used in this study were previously used for verification in other studies as well.^(^ ^22^ ^,^ ^23^ ^)^

As far as gel dosimetry is concerned, cost of readymade commercially available gel with a cylindrical container is around $400 to $500, and cost of the verification software would be not less than $5000 to $6000 or may be more. We have shown a 3D verification methodology using our phantom which will not cost more than $100 for a single verification.

A single big glass or acrylic cylinder can simulate the gel measurement, but simultaneously making measurements with other dosimeters such as ion chamber, film, and TLD is not feasible with such containers and moreover fixation of stereotactic frame in those containers is a tedious process.

**Figure 14:**
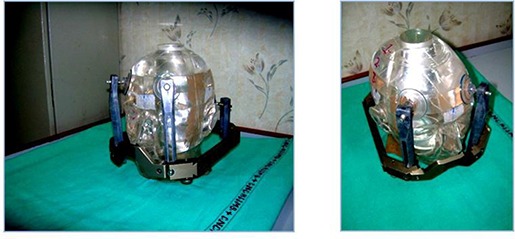
The acrylic phantom fixed in Leksell stereotactic frame with carbon posts.

Newly designed multipurpose acrylic phantom overcomes these issues. It is capable of performing 2D and 3D measurements. This phantom is not only useful for IMRT measurements, but it can be easily fixed in stereotactic frame; hence, it is capable of performing verification measurements in SRS, as well. As we mentioned earlier, we designed this phantom according to our institute QA needs. Figure 14 represents the phantom fixed in Leksell frame (Elekta, Stockholm, Sweden), which is capable of simulating treatment process done for any patient. Measurements with phantom for SRS study will be shown in our future studies. Phantom's inner cylindrical insert (gel) is highly capable of measuring gel dose, but its design is to be reconsidered in future, specifically its gel measurement volume which is beyond the scope of this work for measuring dose for OAR and large tumors. At present it has high potential for verifying SRS and IMRS treatment plans. It proves to be excellent tool for performing film QA study. Since the study is mainly focused on gel measurement, dosimeter which has high spatial resolution (i.e., film dosimeter) was used for comparison, though ion chamber and TLD measurements can also be performed with this phantom, which we will be demonstrating in future studies.

## V. CONCLUSIONS

A novel multipurpose phantom was designed and fabricated using acrylic material for 3D dosimetry. In‐house computer programs written on the MATLAB platform were used for the analyses. Result in this study show that this versatile water equivalent phantom is highly capable for dosimetric verification of conformal treatments. In this work it is also shown that the phantom is suitable for 2D and 3D measurements. Film measurement done with the phantom gave reasonable results, passing all the gamma pass criteria. For the gel measurement, a comprehensive 3D comparison was done between the calculated TPS dose and measured gel dose. Voxel by voxel analysis showed a reasonable agreement. Due to complicated dose distribution and highly modulated nature in IMRT, routinely used 1D and 2D dosimetry may have difficulty to detect these variations at all regions. In this study we have shown methods to extract 3D dosimetric information through gel dosimetry, which shows potential for routine clinical practice.

## ACKNOWLEDGMENTS

We gratefully acknowledge AERB, India for its support through project No. N‐964. Some of the results in the current article were previously presented at the AAPM annual meeting (Pennsylvania, PA) in July 2010 and at the 6th International Conference on 3D Radiation Dosimetry, IC3DDOSE 2010, held at Hilton Head Island, SC, USA.
